# Cortical activation during cooperative joint actions and competition in children with and without an autism spectrum condition (ASC): an fNIRS study

**DOI:** 10.1038/s41598-022-08689-w

**Published:** 2022-03-25

**Authors:** Wan-Chun Su, McKenzie Culotta, Daisuke Tsuzuki, Anjana Bhat

**Affiliations:** 1 Department of Physical Therapy, University of Delaware, 540 S College Avenue, Newark, DE USA; 2 Biomechanics and Movement Science Program, University of Delaware, Newark, DE USA; 3 Department of Language Sciences, Tokyo Metropolitan University, Tokyo, Japan; 4 Department of Psychological and Brain Sciences, University of Delaware, Newark, DE USA

**Keywords:** Cooperation, Predictive markers

## Abstract

Children with an Autism Spectrum Condition (ASC) have social communication and perceptuomotor difficulties that affect their ability to engage in dyadic play. In this study, we compared spatio-temporal errors and fNIRS-related cortical activation between children with and without an ASC during a Lincoln Log dyadic game requiring them to play leader or follower roles, move in synchrony or while taking turns, and move cooperatively or competitively with an adult partner. Children with an ASC had greater motor, planning, and spatial errors and took longer to complete the building tasks compared to typically developing (TD) children. Children with an ASC had lower superior temporal sulcus (STS) activation during Turn-take and Compete, and greater Inferior Parietal Lobe (IPL) activation during Lead and Turn-take compared to TD children. As dyadic play demands increased, TD children showed greater STS activation during Turn-take (vs. Synchrony) and Compete (vs. Cooperate) whereas children with an ASC showed greater IPL activation during Lead and Compete (vs. Cooperate). Our findings suggest that children with an ASC rely on self-generated action plans (i.e., increased IPL activation) more than relying on their partner’s action cues (i.e., reduced STS activation) when engaging in dyadic play including joint actions and competition.

## Introduction

Autism Spectrum Condition (ASC) is a prevalent neurodevelopmental diagnosis affecting 1 in 44 children^1^. Children with an ASC have primary impairments in social communication, including poor social reciprocity, verbal/nonverbal communication skills, as well as the presence of restricted and repetitive behaviors^2^. Apart from the primary symptoms of an ASC, they also present with sensorimotor comorbidities, such as movement incoordination and dyspraxia (i.e., difficulties in performing complex motor sequences)^3–6^, as well as poor executive functions, including poor attention shifting, working memory, response inhibition, and mental flexibility^7^. Together, these primary and comorbid impairments contribute to their difficulties during dyadic play (i.e., play between two individuals).

Dyadic games are a range of simple to complex social interactions that often require different partner roles (as leaders or followers), involve moving with different temporal demands (in-synchrony or when taking turns), and involve common or different goals (playing cooperatively using common goals or competitively using different goals). Dyadic play involving coordination of behaviors between partners (over space and time) to accomplish common goals has been termed joint action^8^. Such rich contexts with varying levels of complexity provide opportunities for motor learning and help build social connections with others^8^. Difficulties in dyadic play especially joint actions could affect children with an ASC’s abilities to learn new skills and establish/maintain social relationships^5,9^. Although studies have posited neural mechanisms underlying social interaction difficulties of children with an ASC, cortical activation patterns during different types of dyadic play including joint actions are not well understood. In the present study, we used a Lincoln Log-based dyadic game that required participating children to build a 3D arrangement of color-coded logs based on a visual card (see Supplementary Fig. S1 for exemplar creations). We compared spatio-temporal errors, hand use, and functional near-infrared spectroscopy (fNIRS)-based cortical activation while children with and without an ASC played leader/follower roles (Lead vs. Follow), moved in synchrony, or while taking turns (Lead, Follow vs. Turn-take), and moved cooperatively or competitively with an adult partner (Lead, Follow, and Turn-take vs. Compete).

### Roles during joint actions—leader or follower

Individuals play different roles during everyday joint actions by being a leader or a follower. For example, musicians follow the cues from the conductor to achieve musical harmony whereas the conductor leads and directs the orchestra. Based on their roles within cooperative actions, individuals apply different movement strategies. Leaders tend to focus on internally driven behaviors, including planning and monitoring of one’s own movements, whereas followers tend to perform externally driven behaviors, such as being socially aware and adjusting to the leader’s actions^10^. Specifically, with the goal of moving together, it is said that leaders often reduce their movement variability so that their movements are more predictable, while the followers prioritize reducing the timing gaps between their own and the leader’s actions^10^.

Multiple functional magnetic resonance imaging (fMRI) studies in healthy participants suggest potential neural mechanisms that support the aforementioned behavioral strategies when playing leader and follower roles. Using a mutually adaptive tapping synchrony paradigm, Fairhurst et al. found greater cortical activation in regions that are important for self-initiated movements, including supplementary motor area, premotor cortex, precuneus, and inferior parietal sulcus, in leaders compared to the followers^11^. When engaging in bimanual movement synchrony using haptic inputs, leaders showed more activation over the primary somatosensory, motor, supplementary motor, as well as dorsolateral prefrontal cortices/middle frontal gyrus (MFG), which are important for motor control and motor planning, while followers showed more activation over the temporoparietal junction and superior temporal sulcus (STS), a part of the mentalizing and social network^12^. Similarly, an fNIRS study found greater activation in temporoparietal and sensorimotor regions when musicians played the second violin part as followers compared to when they played the first violin part as leaders^13^. Taken together, greater cortical activation over the sensorimotor or prefrontal cortices in the leaders may reflect efforts in controlling and planning their own actions, while the greater temporoparietal activation in the followers may reflect their efforts to adapt to partners, to monitor and infer their partner’s actions, and to match their own actions to that of their partner’s. In the present study, we compared behaviors and cortical activation patterns between Lead and Follow conditions during a Lincoln Log dyadic game.

### Temporal components during joint actions—synchrony or turn-taking

Besides different roles, movement timing is also critical to achieve movement goals and ensure appropriate social interactions. While interpersonal synchrony is important for many cooperative tasks, such as moving a heavy object together, turn-taking is embedded into many everyday activities, such as while playing games (i.e., board games) and when engaging in back-and-forth conversations. Both synchrony and turn-taking require one to monitor cues from their social partner, anticipate/predict partner’s movements, and adjust one’s own movements accordingly, therefore, the systems that support perceptuo-motor integration are of particular importance^14^. In contrast to turn-taking, interpersonal synchrony involves moment-to-moment synchronization and the effort of online monitoring and adjustments^14^. Turn-taking, on the other hand, requires one to remember their partner’s actions, wait for one’s turn, and plan one’s own actions; therefore, processes involving working memory, inhibitory control, and motor planning will be important^15^.

Many neuroimaging studies suggest an important role for the observation-execution matching systems (OEMS), including inferior frontal gyrus (IFG), superior temporal sulcus (STS), and inferior parietal lobe (IPL), in matching movements with observed actions; a critical component in synchronous actions or turn-taking^16,17^. The STS region is reported to be more active during movement imitation compared to passive observation or execution, therefore, is said to represent visuomotor correspondences between one’s own and another’s actions^18^. The frontoparietal connections are important for multisensory integration and perceptuomotor control during joint actions^19^. Specifically, the IFG region is important for goal understanding and inferring intentions of observed actions while the IPL region is important for predicting and planning the kinematics of goal-directed actions^20–22^. Other important brain regions include the pre- and post-central gyrus (PCG) and the prefrontal cortices/MFG. In PCG we include the primary motor and somatosensory cortices that receive/process sensory information and execute actions^23^. The prefrontal regions, of such as the MFG and IFG, are important for executive functions such as motor planning, working memory, cognitive shifting, and inhibition—a set of mental skills that are important during interpersonal synchrony and turn-taking^24^.

Using fNIRS, we have reported greater activation over the IFG, STS, and IPL regions in healthy adults and children during interpersonal synchrony compared to solo conditions during reaching and postural sway tasks^25–28^. Similarly, during turn-taking while having conversations or when playing piano duets, healthy adults showed differential frontotemporal activation suggestive of greater social monitoring^29,30^. During a table setting task, adults showed greater IPL activation during turn-taking with another partner vs. moving solo or when observing their partner’s actions^31^. In the current study, we compared behaviors and cortical activation during a naturalistic, Lincoln Log dyadic game involving interpersonal synchrony (Lead and Follow conditions) and turn-taking (Turn-take condition) in children with and without an ASC.

### Intentions during joint actions—cooperative or competitive

Cooperation and competition are important social behaviors for humans. When engaging in cooperative tasks, social partners work towards a shared goal to improve their group performance^32,33^. In contrast, during competitive tasks, the competitors focus on individual goals and would either optimize one’s own performance or undermine their competitor’s performance^34,35^. For both cooperative and competitive behaviors, it is important for one to consider/refer to their partner’s intentions^33,34^. Bratman posits that during cooperation, partners must be aware of each other’s intentions or what has been jointly decided^33^. However, when competing each person in the dyad is only aware of the other’s motor intention or intention-in-action (for example, in the Lincoln Log game, each partner needs to know how far along the other person is to move faster and finish first in the game)^33^. From a neural standpoint, socially-related brain regions such as the bilateral temporoparietal junction and the inferior frontal/prefrontal cortices will be important in both cooperation and competition to monitor partner’s behaviors and to understand the goals and intentions of their actions^35^.

Using a computerized pattern-building game, an fMRI study found common activation over the frontoparietal network during cooperative and competitive behaviors, however, greater orbitofrontal activation was found during cooperative, while greater IPL and medial frontal activation was found during competitive behaviors^36^. Similarly, Liu et al. found fNIRS-related differential activation in the right IFG during competitive and cooperative disc-building games^37^. Using hyperscanning techniques (i.e., simultaneous scanning of partners), the same research group found significant interbrain neural synchronization over right STS during cooperative and competitive conditions, as well as greater right IPL activation during the competitive condition^38^. These results support differential activation of IFG for intention understanding during both competitive and cooperative behaviors, and competition-specific increases in IPL activation to support planning of self-initiated actions and self-other distinctions. In the present study, we compared behaviors and cortical activation during Lincoln Log-based cooperative (Lead and Follow and Turn-take) and competitive (Compete) conditions in children with and without an ASC.

### ASC related difficulties in joint actions

Children with an ASC have poor perceptuomotor control, executive functioning, and intention understanding, that might lead to difficulties in various types of joint actions^2–7,9^. During a joint improvisational mirroring game that required participants to take lead or follow the leader, children with an ASC spent less time in synchrony with their partner, especially when they are in the follower role^39^. They also spent less time synchronizing with the tester during rhythmic actions such as joint marching, clapping, postural sway, and pendulum swaying tasks^3–6,9^. These difficulties have been attributed to their poor visuomotor and inter-limb coordination within solo and social contexts^5,6,9^. Children with an ASC also showed difficulties during turn-taking tasks^40,41^. During back-and-forth conversations, children with an ASC showed longer turn-taking gaps and reduced temporal variability, suggesting poor response inhibition/executive functioning^40^. During cooperative actions, children with an ASC may have difficulties inferring intentions of others which might affect their joint action performance^41^. Salice and Henriksen implicated abnormal joint intentionality as well as we-intentionality in individuals with an ASC^42^. Joint intentionality problems include difficulties perceiving partners’ intentions through deliberation and planning and we-intentionality problems make it difficult for them to perceive themselves as group members and to adopt the group’s perspective. Together, these problems could make it challenging for children with an ASC to socially connect with peers and caregivers^42^.

In terms of cortical activation, children with an ASC have atypical activation over the regions important for OEMS (including IFG, STS, and IPL), executive functioning (including the prefrontal cortices/MFG), and intention understanding (including temporoparietal junction/STS and prefrontal cortices) that might reflect their difficulties in engaging in different types of dyadic play^17,43,44^. Most fMRI studies have investigated ASC-related cortical activation when the participants imitated/followed others finger/hand motions and reported atypical activation over OEMS regions^16,17^. Using fNIRS, our research group has also reported hypoactivation in the IFG and STS regions along with hyperactivation in the IPL region when children with an ASC engaged in synchronous reaching or whole-body sway motions while following the lead of an adult partner^27,28^. Although activation differences between leading and turn-taking joint actions are not well-studied; studies have found reduced prefrontal activation in individuals with an ASC during executive functioning tasks requiring inhibition control and motor planning^43^. Such atypical prefrontal activation might also present in children with an ASC during leading and turn-taking because these tasks require significant motor planning and response inhibition. A lone hyperscanning study involving children with and without an ASC performing cooperative and competitive actions with their parents/strangers found no differences in interpersonal neural coherence even if there was lower behavioral synchrony in children with an ASC versus those without an ASC^45^. Although previous neuroimaging studies have posited neural mechanisms underlying social interaction difficulties of children with an ASC, cortical activation patterns during dyadic play including joint action and competition need further study. The current fNIRS study aims to investigate the ASC-related differences in Cooperative actions (Lead, Follow, Turn-take) and Competition. Specifically, we used a Lincoln Log game that incorporates conditions with different partner roles (Q1: Lead vs. Follow), with different movement timing requirements (Q2: Synchrony-Lead and Follow vs. Turn-take), and with/without shared goals (Q3: Cooperate including Lead, Follow, and Turn-take vs. Compete) to investigate the ASC-related differences as well as condition-related differences in children with and without an ASC. We hypothesized that children with an ASC will show greater spatio-temporal errors and differences in cortical activation over the OEMS (i.e., IFG, STS, IPL) and prefrontal cortices (MFG) for all dyadic play conditions compared to TD children. Moreover, the condition-related differences in children with an ASC will differ from that of TD children. Specifically, we expected differences in cortical activation between (Q1: Lead vs. Follow; Q2: Synchrony (Lead and Follow) vs. Turn-take; Q3: Cooperate (Lead, Follow, and Turn-take) vs. Compete).

## Results

### Differences in behavioral errors, time to completion, and hand preference

**Figure 1:**
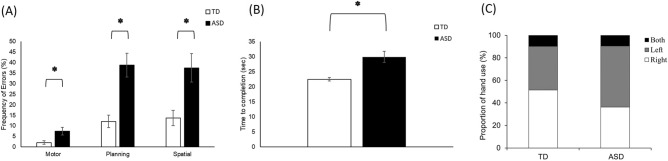
Behavioral errors (**A**), Time to completion (**B**), and Hand preference (**C**) in children with an ASC and TD children. *Indicates significant differences (*p* < 0.05 and survived FDR correction) between ASC and TD groups.

Children with an ASC had greater motor errors [*p* = 0.03; Hedge’s g = − 0.74 (95% CI = − 1.48 to 0.00)], planning errors [*p* = 0.01; Hedge’s g = − 2.32 (95% CI = − 3.25 to − 1.40)], and spatial errors (*p* = 0.03; Hedge’s g = − 1.59 (95% CI = − 2.42 to − 0.77) compared to TD children (Fig. 1A and Supplementary Table S1). Children with an ASC also took longer to complete various dyadic play tasks compared to TD children (*p* < 0.001; Hedge’s g = − 1.24 (95% CI = − 2.02 to − 0.46), Fig. 1B and Supplementary Table S1). Hand preferences did not differ between the two groups as indicated by similar proportions of log pickups using right, left, or both hands (*p* > 0.05, Fig. 1C).

### Cortical activation differences

Repeated measures ANOVAs with the condition, hemisphere, and region of interest (ROI) as within-subject factors, group as a between-subjects factor, and covariates of sex, motor performance (using Bruininks–Oseretsky Test of Motor Proficiency-2 Manual Dexterity scores or BOT-2 MD), and hand preference (i.e., the proportion of right-hand movements) were conducted for averaged oxygenated hemoglobin (HbO_2_) and deoxygenated hemoglobin (HHb) concentration data.

**Figure 2:**
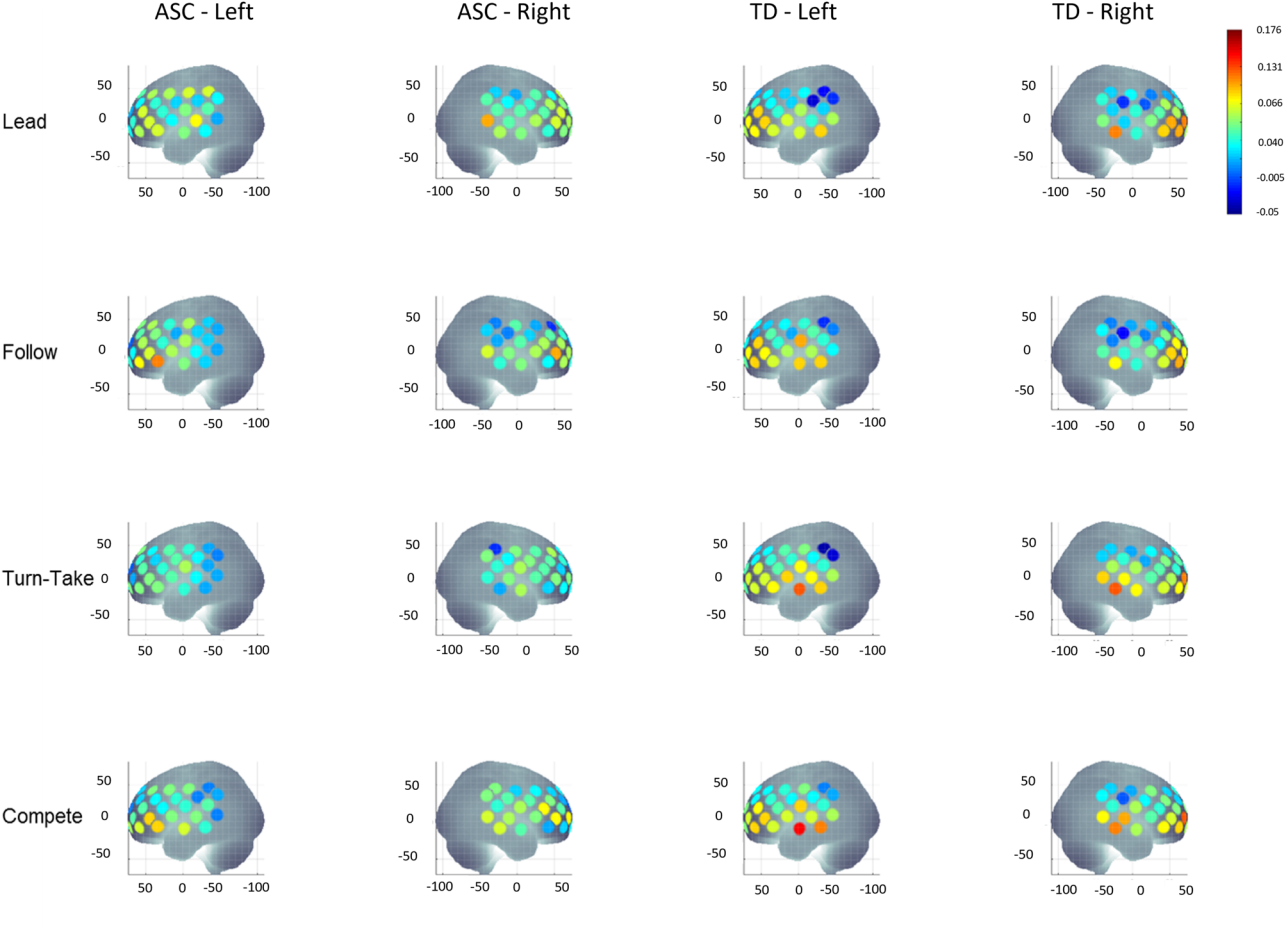
A visual representation of averaged HbO_2_ concentration during Lead, Follow, Turn-take, and Compete conditions in children with an ASC and TD children. HbO_2_ values on Y-axis range from − 0.05 indicated by blue to 0.176 indicated by red. TD children showed greater STS-related channel activation during Compete and Turn-take conditions compared to that of children with an ASC. In contrast, children with an ASC had greater activation in IPL channels during the Lead, Turn-Take, and Compete. This figure was made in MATLAB (version 2021b, https://www.mathworks.com).

For averaged HbO_2_ concentration, a main effect of ROI [F(3.0, 348.2) = 3.0, *p* = 0.030], 2-way interactions of ROI × group [F(3.0, 348.2) = 5.9, *p* = 0.001], hemisphere × ROI [F(3.5, 416.8) = 3.3, *p* = 0.014], 3-way interactions of condition × hemisphere × group [F(3.0, 339.8) = 2.7, *p* = 0.046], condition × ROI × group [F(9.2,1086.9) = 2.2, *p* = 0.02], and hemisphere × ROI × group [F(3.5,416.8) = 3.0, *p* = 0.024], as well as a 4-way interaction of condition × hemisphere × ROI × group [F(9.8,1155.7) = 2.8, *p* = 0.002] were revealed. The 4-way interaction did not covary with sex, BOT-2 MD scores or hand preference, therefore, it was further explored using post-hoc *t* tests. The visual representation of averaged oxygenated hemoglobin (HbO_2_) concentration during all four conditions in both groups is shown in Fig. 2. The means and standard errors (SE) of HbO_2_ concentrations are presented in Supplementary Table S2, and the *p*-values and the direction of effects for significant post- hoc findings are presented in Supplementary Table S4.

For averaged HHb concentration, most effects covaried with one or more covariates (sex, BOT-2 MD, or hand preference) except the 2-way interaction of condition × group [F(2.7, 316.5) = 6.2, *p* = 0.001]. Due to the lack of sensitivity of HHb concentration data in revealing hemispheric and regional differences, we are mainly discussing the averaged HbO_2_ findings below. The means and standard errors (SE) of HHb concentrations are presented in Supplementary Table S3, the *p*-values and the direction of effects for significant post hoc findings are presented in Supplementary Table S5, and the main findings and post-hoc analyses of the HHb condition × group interaction are presented in Supplementary Fig. S2.

### Group differences in cortical activation

**Figure 3:**
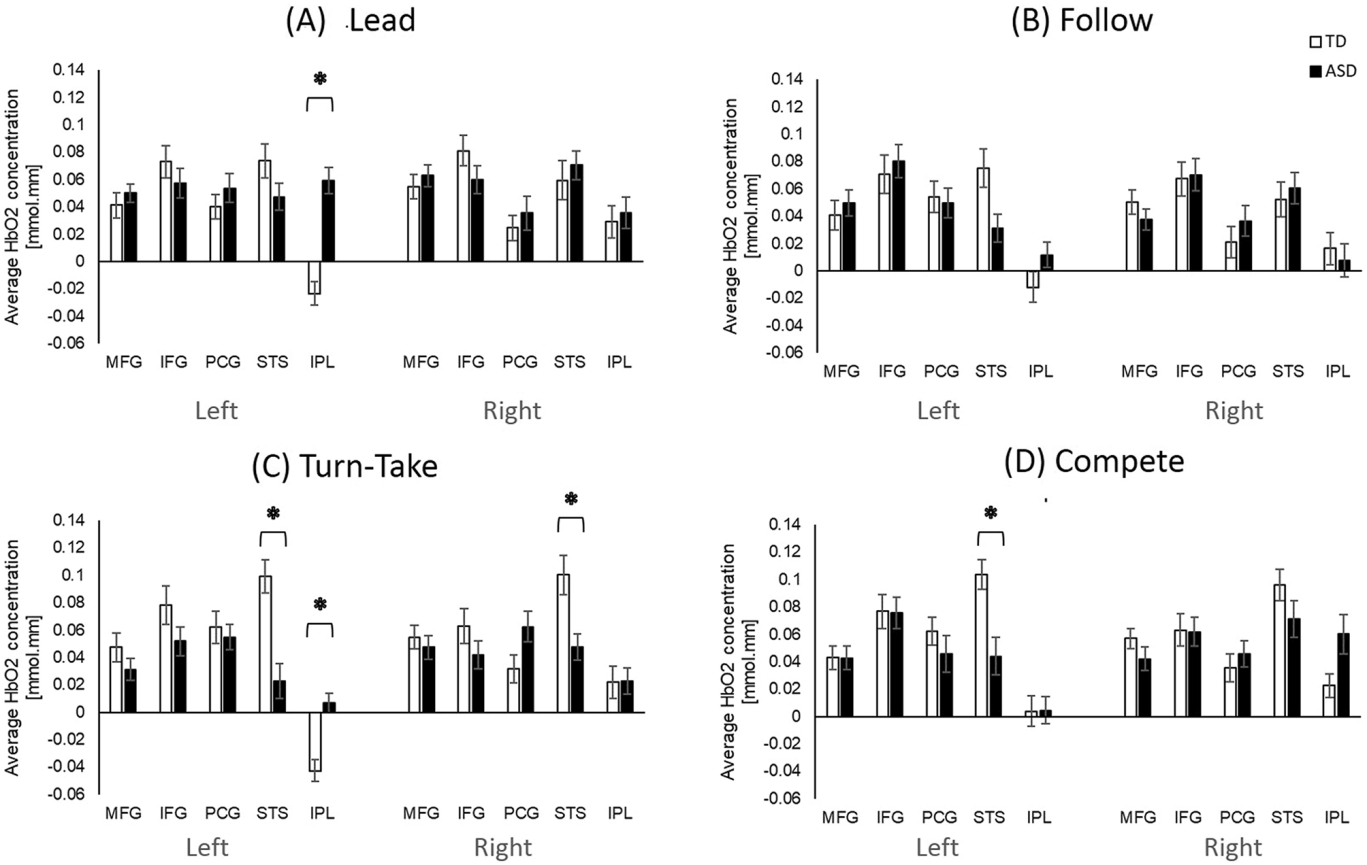
Group differences in cortical activation for Lead (**A**), Follow (**B**), Turn-Take (**C**), and Compete (**D**). *Indicates significant differences (*p* < 0.05 and survived FDR correction) between the ASC and TD groups.

Children with an ASC had lower activation in left and right STS regions during Turn-take (*p*s < 0.01, Hedge’s g = − 0.15 and 0.06 (95% CI = − 0.87 to 0.77); Fig. 3C) and lower left STS activation during Compete compared to the TD children (*p* = 0.001, Hedge’s g = − 0.07 (95% CI = − 0.64 to 0.79); Fig. 3D). They also had lower left STS activation (*p* = 0.01 but did not survive false discovery rate (FDR) correction, Hedge’s g = − 0.26 (95% CI = − 0.98 to 0.46); Fig. 3B) during Follow compared to the TD children. In contrast, children with an ASC had more positive HbO_2_ concentration in the left IPL region during Lead and Turn-take compared to the TD children (*p*s < 0.001, Hedge’s g = − 0.115 and − 0.073 (95% CI = − 0.83 to 0.64); Fig. 3A,C).

### Hemispheric differences in activation

**Figure 4:**
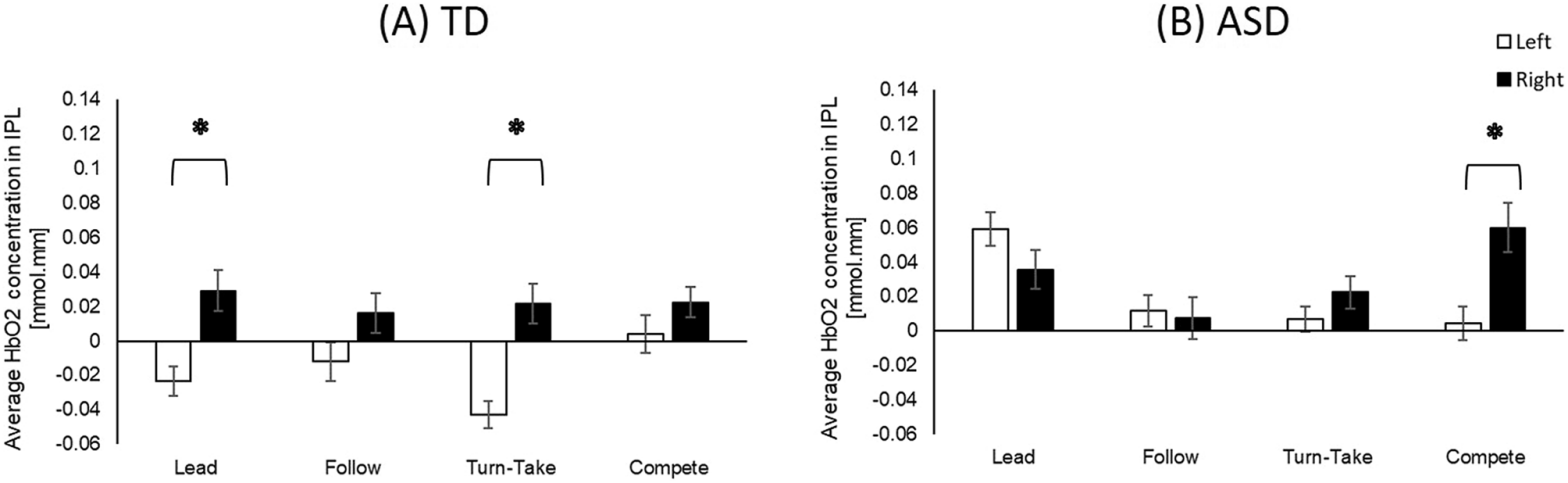
Hemispheric differences between TD children (**A**) and children with an ASC (**B**). *Indicates significant differences between the ASC and TD groups.

For both children with and without an ASC, the hemispheric differences were found only in the IPL region, however, the condition for hemispheric differences differs between groups. Specifically, greater right than left hemispheric activation (i.e., right lateralization) was found in the TD children during Lead and Turn-take (*p*s < 0.001, Hedge’s g = − 0.07 and − 0.09 (95% CI = − 0.81 to 0.64); Fig. 4A), whereas in the children with an ASC, a similar right lateralization pattern was found during Compete (*p* < 0.001, Hedge’s g = − 0.07 (95% CI = − 0.79 to 0.64); Fig. 4B).

### Condition-related differences in cortical activation

**Figure 5:**
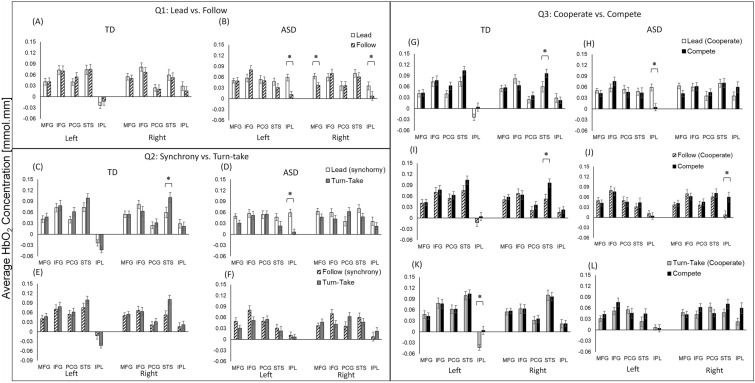
Condition-related differences in cortical activation for TD children and children with an ASC for: (Q1) Lead vs. Follow in TD children (**A**) and children with an ASC (**B**); (Q2) Synchrony (Lead and Follow) vs. Turn-take i.e., Lead vs. Turn-take in TD children (**C**) and children with an ASC (**D**), Follow vs. Turn-take in TD children (**E**) and children with an ASC (**F**); (Q3) Cooperative (Lead, Follow, Turn-take) vs. Compete, i.e., Lead vs. Compete in TD children (**G**) and children with an ASC (**H**), Follow vs. Compete in TD children (**I**) and children with an ASC (**J**), Turn-take vs. Compete in TD children (**K**) and children with an ASC (**L**). *Indicates significant differences (*p* < 0.05 and survived FDR correction) between the ASC and TD groups.

For condition-related differences, we arranged the findings of the post hoc analyses in Fig. 5 based on our initial questions (see the introduction, Q1: Lead vs. Follow Q2: Synchrony-Lead and Follow vs. Turn-take; Q3: Cooperate included Lead, Follow, and Turn-take vs. Compete). For Lead vs. Follow differences*,* the differences were only present in children with an ASC (Figs. 5A,B). Children with an ASC had greater bilateral IPL and right MFG activation during the Lead compared to the Follow condition (*p*s < 0.001, Hedge’s g = 0.03–0.06 (95% CI = − 0.68 to 0.78); Fig. 5B). For Synchrony-Lead and Follow vs. Turn-take, TD children had greater right STS activation during Turn-take compared to Lead (*p* = 0.01, Hedge’s g = 0.54 (95% CI = − 0.19 to 1.26); Fig. 5C), while children with an ASC had greater left IPL activation during Lead compared to Turn-take (*p* < 0.01, Hedge’s g = − 0.07 (95% CI = − 0.65 to 0.79); Fig. 5D). There was no significant difference between Follow and Turn-take in TD children and children with an ASC (no *p*-value survived the FDR corrections, Fig. 5E,F). For Cooperate-Lead, Follow, Turn-take vs. Compete, TD children showed greater right STS activation during Compete than Lead and Follow (*p*s < 0.01, Hedge’s g = 0.54 and − 0.05 (95% CI = − 0.77 to 1.27); Fig. 5G,I). In addition, the TD children showed greater left IPL activation during Compete vs. Turn-take (*p* = 0.001, Hedge’s g = − 0.07 (95% CI = − 0.78 to 0.65); Fig. 5K). Children with an ASC had greater left IPL activation during Lead vs. Compete (*p* < 0.001; Hedge’s g = 0.072 (95% CI = − 0.64 to 0.79); Fig. 5H) and greater right IPL activation during Compete vs. Follow (*p* = 0.0001, Hedge’s g = − 0.062 (95% CI = − 0.78 to 0.65); Fig. 5J). There were no conditional differences between Turn-take and Compete in children with an ASC (*p*-values did not survive FDR corrections, Fig. 5L).

### Correlations between cortical activation and behavioral performance and Vineland Adaptive Behavioral Scales (VABS) scores

Correlations between cortical activation and behavioral errors did not survive FDR corrections (Supplementary Table S6). In terms of relations with VABS, a positive correlation implies greater activation in children with better VABS performance. TD children with higher VABS social scores had greater activation over left STS during Lead (r = 0.449, *p* < 0.001) and greater activation over bilateral MFG, STS, left IPL, and right IFG during Follow (r = 0.309–0.366, *p*s < 0.05; Table 1). TD children with greater VABS Daily living scores had greater left PCG, IPL, right IFG activation during Turn-Take (r = 0.378–0.391, *p* < 0.01), and lower left PCG activation during Compete (r = − 0.387, *p* < 0.01; Table 1). Children with an ASC showed significant correlations between cortical activation and VABS/SRS scores only in the Lead condition. Specifically, children with an ASC with greater VABS Daily living score had greater right IFG activation during Lead (r = 0.465, *p* < 0.001; Table 1).

**Table 1:** Correlations between cortical activation and the VABS and SRS scores in children with and without an ASC.

ROIs	TD group				ASC group			
	VABS_Communication	VABS_Social	VABS_Daily Living	SRS	VABS_Communication	VABS_Social	VABS_Daily Living	SRS
**Lead**								
Left								
MFG	− 0.155	0.170	− 0.112	− 0.021	0.111	− 0.008	0.082	− 0.298*
IFG	− 0.080	0.021	0.130	0.166	0.124	0.125	− 0.046	− 0.044
PCG	− 0.021	0.198	− 0.022	0.111	− 0.200	− 0.280*	− 0.244	0.035
STS	0.240	0.449**	0.228	0.205	0.179	0.222	0.128	− 0.300*
IPL	− 0.006	0.088	− 0.178	0.210	− 0.090	− 0.190	− 0.117	0.160
Right								
MFG	− 0.140	0.050	− 0.134	− 0.070	− 0.111	− 0.191	0.099	− 0.058
IFG	0.215	0.229	0.215	0.103	0.285*	0.186	0.465**	− 0.453**
PCG	− 0.175	− 0.041	− 0.334**	− 0.054	0.185	0.170	0.283*	− 0.248
STS	0.261*	0.314*	0.216	0.118	0.129	− 0.040	0.109	− 0.359**
IPL	− 0.218	0.029	− 0.271*	0.284*	0.329*	0.313*	0.192	0.018
**Follow**								
Left								
MFG	0.145	0.307*	0.179	− 0.108	0.067	− 0.033	− 0.093	− 0.124
IFG	0.034	0.079	0.117	− 0.140	− 0.112	− 0.084	− 0.275*	0.153
PCG	− 0.008	0.140	− 0.172	− 0.099	0.202	0.222	0.134	− 0.283*
STS	0.193	0.345**	0.105	− 0.033	0.019	0.050	− 0.051	− 0.072
IPL	0.290*	0.329*	0.143	0.044	− 0.157	− 0.088	− 0.156	0.256
Right								
MFG	0.128	0.309*	0.105	− 0.161	− 0.087	− 0.115	− 0.144	− 0.009
IFG	0.235	0.366**	0.350**	0.085	− 0.079	− 0.055	0.031	0.033
PCG	0.219	0.164	0.045	− 0.145	0.194	0.094	0.170	− 0.103
STS	0.135	0.353**	0.149	0.033	− 0.048	− 0.009	− 0.053	0.155
IPL	− 0.158	0.691	− 0.115	0.020	0.044	0.052	− 0.020	0.236
**Turn-Take**								
Left								
MFG	0.062	0.183	0.219	0.077	− 0.131	− 0.174	− 0.239	0.067
IFG	− 0.053	0.011	0.036	0.051	0.150	0.133	− 0.016	− 0.015
PCG	− 0.090	0.043	0.375**	0.061	0.027	− 0.094	0.073	0.076
STS	0.143	0.237	− 0.226	− 0.259*	0.132	0.133	− 0.022	− 0.059
IPL	0.152	0.147	0.391**	0.143	− 0.144	− 0.083	0.010	0.011
Right								
MFG	− 0.038	0.188	0.170	0.022	− 0.341*	− 0.317	− 0.263	0.241
IFG	0.334**	0.313*	0.385**	0.036	0.211	0.038	0.238	− 0.382**
PCG	0.001	0.077	− 0.085	0.0003	0.162	0.031	0.079	− 0.004
STS	0.198	0.164	− 0.023	− 0.228	0.283*	0.234	0.076	− 0.041
IPL	0.014	0.260*	0.105	0.124	0.168	0.050	0.066	− 0.010
**Compete**								
Left								
MFG	− 0.031	0.072	0.020	0.071	0.083	− 0.056	− 0.093	− 0.073
IFG	− 0.027	0.103	0.115	0.186	− 0.056	− 0.125	− 0.207	0.134
PCG	0.002	0.234	− 0.387**	0.028	0.108	0.111	0.071	− 0.048
STS	0.147	0.023	− 0.064	− 0.119	0.074	0.066	− 0.095	− 0.107
IPL	− 0.020	0.066	− 0.243	0.102	0.156	0.112	0.176	0.132
Right								
MFG	− 0.049	0.133	0.164	0.060	0.005	0.008	0.123	− 0.053
IFG	0.250	0.281*	0.141	− 0.075	− 0.094	− 0.069	0.006	− 0.042
PCG	0.101	0.201	− 0.085	0.051	0.165	0.165	0.035	− 0.051
STS	0.233	0.257*	0.058	− 0.017	0.116	0.049	0.021	− 0.160
IPL	− 0.039	− 0.040	− 0.052	0.147	0.223	0.287*	0.280*	0.217

*p < 0.05; **p < 0.01. Bolded font indicates *p*-values survived for FDR corrections.

### Correlation between Cortical activation and Social Responsiveness Scale (SRS) scores

No significant correlations were found between cortical activation and SRS scores in the TD group (Table 1). Higher SRS scores indicate poor social performance, hence, a negative correlation implies lower activation in children with poor social responsiveness. During Lead, children with an ASC with poor social performance had lower right PCG, and STS activation (r = − 0.359 to − 0.453, *p*s < 0.01). During Turn-Take, children with an ASC with poor social performance had lower right IFG activation (r = − 0.382, *p*s < 0.01, Table 1).

## Discussion

Previous fMRI studies of dyadic interactions/joint actions have been limited to simple hand movements and unnatural environments. Most studies focused on imitation and synchrony-based cooperative actions but not turn-taking or competition. Using fNIRS and motion tracking systems, we have reported differences in behavioral performance and cortical activation in healthy adults and school-age children with and without an ASC during multiple interpersonal synchrony tasks involving reaching/body sway versus solo actions^25–28^. In this study, we extend our past work to a novel naturalistic, dyadic building game using Lincoln Logs in children with and without an ASC. We found that children with an ASC had greater motor, planning, and spatial errors, and they took longer to complete the tasks compared to the TD children. For group-based activation differences, children with an ASC had lower bilateral STS activation during Turn-take, and lower left STS activation during Compete, and a similar statistical trend for Follow compared to TD children. In contrast, children with an ASC had greater left IPL activation during Lead and Turn-take compared to TD children. For hemispheric differences, TD children had right lateralized IPL activation during Lead and Turn-take, whereas children with an ASC had right lateralized IPL activation during Compete. For condition-related differences, TD children had a consistent pattern of greater right STS activation during dyadic play tasks involving greater social monitoring/intention inferring demands (Turn-take > Lead, Compete > Lead, and Compete > Follow). They also had greater left IPL activation during Compete vs. Turn-take. In contrast, children with an ASC had a completely different strategy of greater left and/or right IPL or right MFG activation during Lead vs. Follow, Lead vs. Turn-take, Lead vs. Compete, and Compete vs. Follow. For correlations between the cortical activation and the adaptive functioning measure in both groups, better VABS socialization and/or daily living performance was associated with greater cortical activation. For correlations between cortical activation and SRS scores, children with an ASC’s poor social performance was also associated with lower right IFG, and/or STS activation during Lead and Turn-Take conditions.

We found that children with an ASC had greater motor, spatial, and planning errors and took longer to complete the tasks compared to their TD peers. Children with an ASC have poor social awareness, visuo-motor coordination, and executive functioning skills, which might affect their joint building abilities^46–49^. Poor social monitoring is a fundamental diagnostic impairment and is widely reported in children with an ASC. Children with an ASC are less likely to follow an adult partner’s gaze or gestural bids to observe objects in the environment^46,49^. Toddlers with an ASC who were shown 2D clips of a complex scene involving objects and people pay less attention to interacting adults and paid more attention to the surrounding background^50^. Moreover, dyspraxia (i.e., difficulties performing skilled motor sequences) is often reported in children with an ASC with greater spatio-temporal errors and greater time to task completion compared to those without an ASC^3–6^. Together, these motor coordination/planning and social impairments could impair dyadic play performance in children with an ASC.

Children with an ASC had lower bilateral STS activation during Turn-take and lower left STS activation during Compete and Follow compared to TD children. In contrast, they had greater left IPL activation during Lead and Turn-take than the TD children. Recent studies have reported that followers showed greater STS activation whereas leaders showed greater supplementary motor area and sensorimotor activation during synchronous movement or playing of the violin^12,13^. When planning joint actions, children with an ASC may rely more on internal or self-generated plans than being externally driven (i.e., using social information from their partners to plan their actions). For hemispheric differences, TD children had right lateralized IPL activation during Lead and Turn-take conditions whereas children with an ASC had a similar right lateralized IPL activation during the Compete condition. Right IPL is important in making self-other distinctions when engaging in synchrony or competition vs. cooperation tasks^36,51^. Less right IPL activation is expected when cooperating as it may involve greater merging of self and other whereas greater right IPL activation is expected when competing with a partner^36^. In the present study, children with an ASC also showed a similar pattern of greater right lateralized IPL activation during Compete vs. Cooperate. Interestingly, TD children showed a similar pattern of greater right-lateralized IPL activation in Lead and Turn-take; this may also be attributed to greater deactivation in left IPL across multiple joint action conditions. The left IPL region is considered part of the Default Mode Network (DMN) and is said to deactivate when performing externally directed processing (i.e., tasks that are cognitively demanding, goal-directed, or requiring greater social awareness)^28,52^. The DMN is said to be important during social as well as imitation tasks^53^. In fact, being imitated led to greater DMN deactivation, compared to when imitating others suggesting that an individual is perhaps more socially aware of their partner’s actions when they are able to regulate the social interaction^54^. Consistent with this finding, TD children showed more deactivation in the left IPL across multiple joint action tasks whereas such left IPL deactivation was rarely seen in children with an ASC. Past studies have also reported a lack of DMN deactivation in children with an ASC during other cognitively demanding tasks^28,55,56^.

For most dyadic play comparisons, TD children had a consistent pattern of greater right STS activation during tasks involving greater social monitoring/intention inferring demands (Turn-take vs. Lead, Compete vs. Lead, and Compete vs. Follow). They also had reduced deactivation in the left IPL during Compete vs. Turn-take suggesting that they were more internally driven when Competing vs. when Cooperating/Turn-taking. In contrast, children with an ASC had greater bilateral IPL and right MFG activation during Lead vs. Follow, greater left IPL activation during Lead vs. Follow, Lead vs. Turn-take, and Lead vs. Compete, and greater right IPL activation during Compete vs. Follow. Taken together, TD children had greater activation in an important social network region (i.e., the STS) suggesting they may be more socially aware during tasks involving greater social monitoring/intention inferring demands (i.e., Follow, Turn-take, and Compete vs. Lead). In addition, they might utilize more internally driven/self-initiated planning when competing as seen by reduced left IPL deactivation.

In contrast, children with an ASC seem to use a completely different IPL-based strategy wherein they showed reduced deactivation and greater left IPL activation during Lead compared to other joint action conditions, indicating greater reliance on self-generated action plans. Lastly, they also showed greater right IPL activation during Compete vs. Cooperate. Taken together, children with an ASC may engage in better self-other distinctions when Leading and Competing with partners compared to Following and Cooperating with others making Leading and Competition valuable learning/intervention contexts to promote greater self vs. other awareness.

For correlations between activation and the adaptive functioning in both groups, better VABS social, and daily living performance was generally associated with greater activation over multiple cortical ROIs. For correlations between cortical activation and SRS scores, children with an ASC that had better social performance had higher right IFG, and/or STS activation during Lead and Turn-Take conditions. During joint actions, one must anticipate their partner’s actions by observing them, infer their intentions, plan one’s own actions, and execute the action plan^14^. The networks formed by MFG, IFG, PCG,STS, and IPL regions are implicated in each of these processes (i.e., social awareness (STS), intention inferring (IFG), planning actions (MFG and IPL), and action execution (PCG) along with many other important brain regions).

Although several significant and meaningful findings were revealed, the current pilot study involved a small sample size, was not perfectly matched for sex distribution between groups and included a broad range of functioning. The small sample size might lead to greater variability and smaller effects (Hedge’s g = − 0.26 to 0.04) and limited our ability to conduct subgroup analyses. Small effect sizes are often reported in neuroimaging studies involving children with an ASC^17^, and hence, we ask readers to interpret our results with caution. We have controlled for differences in sex distribution across groups by adding sex as a covariate in the ANOVA. We also chose a less conservative statistical correction approach, which is more inclusive of findings and report differences that need to be further investigated by larger future studies. While we followed consistent probe placement, variation in participant head sizes and probe placement could have led to inconsistency in our spatial registration output.

## Conclusions

Our study identified multiple potential behavioral and fNIRS-based neurobiomarkers during a Lincoln Log-based joint action game across prefrontal, frontal, temporal and parietal cortices. Children with an ASC had greater behavioral errors (motor, spatial, and planning) and took greater time to complete tasks. In addition, children with an ASC showed reduced STS activation and increased IPL activation as well as a lack of differential activation in the STS region compared to TD children. We also found different patterns of activation in children with an ASC compared to TD children suggesting that both groups used different mechanisms to process social-perceptual information for motor planning/execution of joint actions. Currently, we are conducting a randomized controlled trial in children with ASD to further validate fNIRS-related synchrony-based neurobiomarkers as intervention response measures following a bout of socially-embedded intervention focused on imitation, synchronization, and cooperation. Overall, fNIRS appears to be a valid and powerful child-friendly tool to examine cortical activation during joint play in both children with and without an ASC. From a learning standpoint, clinicians must consider utilizing opportunities for leading and competition to improve social awareness in children with an ASC apart from following and cooperation. Future studies must build on the potential fNIRS biomarkers identified in the present study to assess their reliability, scalability, consistency, and replicability across sites and studies.

## Methods

### Participants

Thirty children with and without an ASC (Average ± SE: ASC: 11.5 ± 0.8, 12 males, 3 females; TD: 12.2 ± 0.9, 8 males, 7 females) between 6 and 17 years participated. The recruitment of the ASC group was slightly ahead of the TD group. We included the TD children who were age-matched (age difference < 1 year) with children in the ASC group that were already recruited. There were no significant group differences in age, sex, or ethnicity (*p*s > 0.05). Children were recruited through online postings, phone calls, and fliers sent to ASC advocacy groups, and Simons Powering Autism Research (SPARK) participant research match service. SPARK informs their family database about research studies (https://www.sfari.org/resource/spark/). Before participation, we completed screening interviews with potential participants to obtain their demographic information and to confirm their eligibility. The inclusion criteria for children with an ASC were (i) should hold a professionally confirmed ASC diagnosis, supported by school records, an Individualized Education Plan for ASC-related services, or medical/neuropsychological records from a psychiatrist or clinical psychologist using the Autism Diagnostic Observation Schedule; there is a growing trend of using professionally confirmed diagnostic records for ASC cohort studies^57^ and (ii) met criteria for a social communication delay (> 12 points) on the Social Communication Questionnaire (SCQ)^58^. Children with an ASC were excluded if they had any behavioral/sensory issues that prevented them from completing the test activities. The age-matched TD children were excluded if they had any neurological or developmental disorder/delay or a family history of an ASC.

Parents of all children completed the Coren handedness survey to assess hand preferences^59^, the VABS measure to assess adaptive functioning^60^, and SRS to assess social responsiveness impairment^61^. Additionally, we administered the BOT-2 MD to assess fine motor skills^62^. Compared to TD children, children with an ASC had significantly lower VABS, BOT-2 MD scores, and greater SRS total scores indicating impaired adaptive functioning, manual dexterity performance, and social responsiveness (Table 2). All study procedures were carried out in accordance with the Declaration of Helsinki. All consent and assent forms as well as all study procedures were approved by the University of Delaware Institutional Review Board (UD IRB, Study Approval #: 930721). Prior to study participation, written informed consent was obtained from parents who gave approval on behalf of their children as their legal guardians and written and verbal assent was obtained from the children. Written parental and experimenter permission/consent to use their pictures for this publication has been taken.

**Table 2:** Demographic information and questionnaires data for ASC and TD groups.

Characteristics	TD group (n = 15) Mean ± SE	ASC group (n = 15) Mean ± SE
Age	12.2 ± 0.9	11.5 ± 0.8
Sex	8M, 7F	12M, 3F
Ethnicity	12C, 2 A, 1 AA	11C, 2A, 2AA
Handedness	14R, 1L	13R, 2L
**VABS (%)**		
Composite Score	67.7 ± 6.0*	4.2 ± 1.3
Communication	65.8 ± 5.7*	4.6 ± 1.3
Socialization	77.4 ± 4.1*	4.8 ± 1.8
Daily Living	74.8 ± 5.8*	8.7 ± 3.4
BOT MD Raw Scores	26.3 ± 1.5*	21.4 ± 1.9
SRS Total score	22.5 ± 4.2*	111.1 ± 7.1

*SE* standard error, *VABS* Vineland Adaptive Behavioral Scale, 2nd edition, *BOT MD* Manual Dexterity subtest of the Bruininks–Oseretsky Test of Motor Proficiency, 2nd edition, *SRS* Social Responsiveness Scale, 2nd Edition, *M* male, *F* female, *C* Caucasian, *A* Asian, *AA* African-American, *R* right-handed, *L* left-handed, *indicates a significant difference between groups (i.e., *p*-value < 0.05).

## Experimental procedures

**Figure 6:**
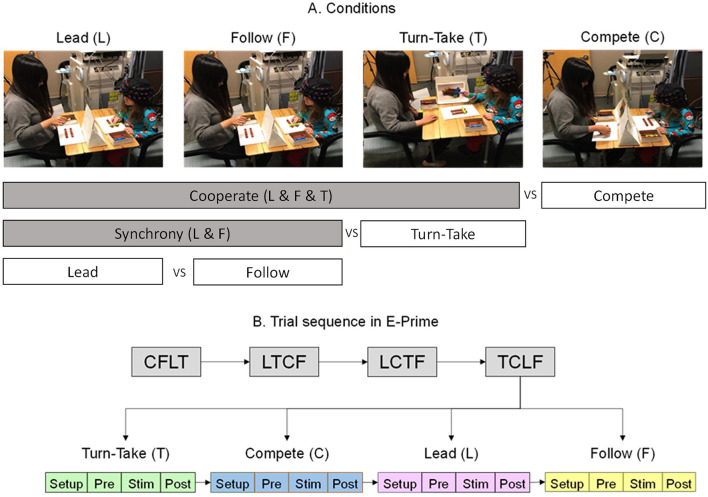
Experimental conditions of Lead, Follow, Turn-take, and Compete (**A**), and the randomized blocked design protocol for the dyadic task (**B**).

Each child sat at a table across from an adult tester and was fitted with a 3 × 11 fNIRS probe set (Fig. 6A). A container of Lincoln logs consisting of four plain brown logs and four multi-colored (green, yellow, purple, blue) supporting logs was placed on the table and a visual cue card was placed facing one or both participants (See Supplementary Fig. S1 for cue card examples). The Lincoln Log game was chosen because it could be easily color-coded and made into simple but variable configurations. It is also less demanding in fine motor skills/strength compared to the more common building games such as Lego, which require more fine motor skills to assemble/disassemble the pieces. Additionally, since Lincoln logs are less common compared to building games such as Lego, it kept the task equally novel across children.

During the Lincoln Log building game, each child/adult partner was asked to build the logs according to the assigned picture card with rules based on four conditions, Lead, Follow, Turn-take, and Compete. In the Lead condition, the child built the configuration according to the visual cue card (shown to the child only) while making sure that the follower/adult tester followed their actions simultaneously. In the Follow condition, the child mimicked the building actions and moved synchronously with the tester who was the only one shown the cue card. In the Turn-take condition, the cue card was visible to both partners. They took turns to make the next move and built the log configurations together. In the Compete condition, non-identical cue cards were given to both, tester and child, and they were asked to quickly and independently build the structure shown on their cue card. We used a randomized block design comprised of 16 trials, i.e., 4 blocks were completed with the 4 conditions presented in a random order (Lead, Follow, Turn-take, Compete; Fig. 6B). Each trial included a 10-s pre-stimulation, 15-s stimulation, and a 15-s post-stimulation period (Fig. 6B). During the pre- and post-baseline periods, participants were asked to observe a crosshair. We were unable to collect more trials because the entire session lasted around 25–30 min, beyond which children experience discomfort due to the weight of the 52-channel fNIRS cap.

### Data collection

The Hitachi ETG-4000 system was used to capture the hemodynamic changes during the joint action tasks (Hitachi Medical Systems, Tokyo, Japan, Sampling Rate: 10 Hz). A cap embedded with a 3 × 11 probe set (including 17 infrared emitters and 16 receivers) was positioned over frontal, temporal and parietal regions of the brain (see Supplementary Fig. S3). The midline of the probe set was aligned with the nasion, and the lower border of the probe set was aligned just above the eyebrow and the ears. The adjacent pairs of probes, located 3 cm apart, acted as emitters and receivers for two wavelengths of light (695 and 830 nm). Light waves travel from the emitter through the skull, creating a banana-shaped arc reaching the capillary bed of the cortical tissue of the brain. Some of the light waves are absorbed/scattered while the remaining waves are transmitted back to the receivers. Using the Modified Beer-Lambert law, change in light attenuation is used to determine changes in the concentration of HbO_2_ and deoxygenated hemoglobin (HHb) at the midpoint of two probes, also termed a channel. When a certain cortical region is more active, there will be an increase of metabolic demand/oxygen consumption and blood flow to the capillary bed supplying that cortical region, which in turn leads to an increase in HbO_2_, and a slight decrease in HHb^63^. E-prime 2.0 software was used to trigger the ETG system and mark the baseline and stimulation periods. The session was videotaped using a camcorder that was synchronized with the ETG-4000 system.

### Spatial registration approach

We recorded the 3D location of standard cranial landmarks (nasion, inion, right/left ear) and each fNIRS probe with respect to a reference coordinate system using a Polhemus motion analysis system. These 3D coordinates were saved in a text file for each participant and later run through MATLAB codes developed by the third author. The anchor-based, spatial registration method developed by Tsuzuki et al.^64^ was used to transform the 3D spatial location of each channel from the reference coordinate system to the Montreal Neurological Institute (MNI)’s coordinate system. Structural information from an anatomical database^65^ was used to provide estimates of channel positions within a standardized 3D brain atlas. The estimated channel locations were anatomically labeled using the LONI Probabilistic Brain Atlas (LPBA)^66^. Note that each run includes position data from all participants across both groups to obtain the average MNI coordinates for each channel. We assigned each channel to a certain ROI if 65% or more of the channel area (i.e., each channel was modeled as the centroid of a sphere) was within a given ROI. Based on these rules, we assigned 38 out of 52 channels to five ROIs in each hemisphere (Ten ROIs in total; See Supplementary Fig. S3 and Table S7) as follows: (i) MFG (right: 3, 4, 14, 15, 25, 36; left: 7, 8, 17, 18, 28, 38); (ii) IFG (right: 24, 34, 35, 45; left: 29, 39, 40, 50); (iii) PCG (right: 2, 13, 23; left: 9, 19, 30); (iv) STS (right: 32, 33, 43, 44; left: 41, 42, 51, 52); (v) IPL (right: 1, 11; left: 10, 21). On average, the variability of placement for channels included was 10.9 ± 0.1 mm. Average HbO_2_/HHb concentration values for a stimulus period were obtained for each channel. Later, the average HbO_2_/HHb values for all channels belonging to the same ROI were averaged.

### Data processing

We have developed custom MATLAB codes that incorporate functions from open-source software including HOMER-2 and Hitachi POTATo to process the fNIRS data output^67,68^. The sampling frequency of the fNIRS system was 10 Hz (i.e., 10 data frames per second were collected). Data from each channel was first band-pass filtered between 0.01 and 0.5 Hz using the Fast Fourier Transform (FFT) method to remove lower or higher frequencies associated with body movements and other dynamic signals/tissue such as heart rate, skin blood flow, etc. The low-pass filter removes physiological noises related to fast cardiac oscillations and high-frequency instrument noise, whereas the high-pass filter minimizes the low-frequency drift from the data. To remove motion artifacts, we used the wavelet method (implemented in the Homer-2 software)^67^. In this method, it is assumed that the measured signal is a linear combination of the desired signal and the undesired artifacts. By applying the 1-D discrete wavelet transform to the signal from each channel, details of the signal are estimated as approximation coefficients. Assuming that the detail wavelet coefficients have a Gaussian distribution, outliers in the distribution correspond to the coefficients related to the motion artifacts and hence such coefficients are set to zero. Lastly, the inverse discrete wavelet transform is applied, and the signal is reconstructed. Next, the General Linear Model (GLM) was implemented using a HOMER-2 MATLAB function. GLM estimated the hemodynamic response function using Gaussian basis functions and a 3rd order polynomial drift regression^67^. To correct the baseline drifts, the linear trend between the pre-trial baseline and the post-trial baseline was calculated and subtracted from values in the stimulation period as implemented in Hitachi POTATo^68^. An average HbO_2_ and HHb values were obtained for the stimulation period of each trial. Both, HbO_2_ and HHb values were analyzed for better physiological interpretation^69^. The range of HbO_2_ data was significantly greater than HHb data and HbO_2_ is said to have a larger signal to noise ratio, hence; is reported within the manuscript. HHb data is shown within the supplementary materials due to its lack of sensitivity as explained earlier under ANOVA descriptions. We plotted and saved data at each step and visually screened the plotted figures at each step of the analysis to exclude channels/trials. We excluded channels with poor contact (flat lines) or persistent motion artifacts or obvious outliers compared to the other similar trials from each condition. Readers may also refer to our earlier publications for details^25–28^.

### Behavioral coding

We established inter-rater reliability between two student coders using 20% of the total videos (inter-rater reliability was > 90%) to confirm that the coding definitions were approved by two individuals. The intra-rater reliability was mainly established for the primary coder only using 20% of the total videos were coded (intra-rater reliability was > 95%). Each session was scored for three error types: (i) Motor error: the child dropped a log before placing in the container or knocked over the container; (ii) Planning error: the child hesitated, and then changed placement location; and (iii) Spatial error: the log was placed incorrectly based on color or location. Furthermore, we coded hand preferences by scoring how the child picked up each log using their left, right, or both hands. Lastly, we coded the time in seconds to complete each building configuration.

### Statistical analyses

Kolmogorov–Smirnov test were used to test the normal distributions of the fNIRS (HbO_2,_ HHb), questionnaire (VABS, SRS), and behavioral performance data (behavioral errors, time to completion, and hand preference). 75% of the fNIRS data were normally distributed, while all of the questionnaires, and behavioral performance data were not normally distributed (*p*s < 0.05) To assess group differences in frequency of behavioral performance (errors of each type, time to completion, and hand preference), we conducted non-parametric, Mann–Whitney *U* tests. For cortical activation (averaged HbO_2_ and HHb), we conducted a repeated measure ANOVA using within-group factors of condition (Lead, Follow, Compete, Turn-Take), region (MFG, PCG, IFG, STS, IPL), hemisphere (Left, Right), a between-group factor of group (ASC, TD) with sex, BOT-2 MD score, and hand preference as covariates. When our data violated Mauchly’s test of sphericity, corrected F-values following Greenhouse–Geisser or Hyunh–Feldt corrections have been reported. Lastly, Spearman correlations were used to correlate cortical activation and behavioral performance (both groups), VABS (both groups) and SRS performance (ASC only). These analyses were conducted through IBM SPSS. To control for multiple comparisons for post-hoc analyses and correlation runs, the Benjamini–Hochberg False Discovery Rate (FDR) correction method was used to adjust the statistically significant cut-off^70^. Specifically, the unadjusted *p*-values were rank ordered from low to high and the statistical significance was declared if the unadjusted *p*-value was less than the *p*-value threshold which was determined by multiplying 0.05 with the ratio of the unadjusted *p*-value rank to the total number of comparisons (*p*-threshold for ith comparison = 0.05 × i/n; where n = number of comparisons). To be clear, a result was considered significant if the obtained *p*-value was less than the FDR corrected *p*-value. To better interpret the results, we further calculated effect sizes for significant post-hoc comparisons using the Hedge’s g method^71^.

## Supplementary Information

Supplementary Information.

## Data Availability

The datasets generated during and/or analyzed during the current study are available from the corresponding author on reasonable request.
