# Supplementary material for: Cortical activation during cooperative joint actions and competition in children with and without an autism spectrum condition (ASC): an fNIRS study
Source: Sci Rep. 2022 Mar 25;12:5177. doi: 10.1038/s41598-022-08689-w (PMC8956636; doi:10.1038/s41598-022-08689-w)

# Cortical Activation during Cooperative Joint Actions and Competition in Children With and Without an Autism Spectrum Condition (ASC) – An fNIRS Study

**Wan-Chun Su,[1,2] McKenzie Culotta,[1,2] Daisuke Tsuzuki,[3] Anjana Bhat,[1,2,4*]**

**Supplementary Table S1.** Means and standard errors (SE) of behavioral errors in children with and without an ASC during Lead, Follow, Turn-take, and Compete conditions of the Joint action task.

| | **Lead** | | **Follow** | | **Turn-take** | | **Compete** | |
|---|---|---|---|---|---|---|---|---|
| | **Mean** | **SE** | **Mean** | **SE** | **Mean** | **SE** | **Mean** | **SE** |
| **TD group** | | | | | | | | |
| Motor error | 0.0 | 0.0 | 1.7 | 1.7 | 1.7 | 1.7 | 5.0 | 2.7 |
| Planning error | 10.0 | 6.8 | 8.3 | 4.7 | 16.7 | 7.2 | 13.3 | 4.8 |
| Spatial error | 6.7 | 6.7 | 30.0 | 8.9 | 11.7 | 7.3 | 6.7 | 4.5 |
| Time to completion | 20.5 | 1.04 | 24.8 | 1.1 | 24.3 | 1.0 | 20.2 | 0.9 |
| ASC **group** | | | | | | | | |
| Motor error | 6.3 | 2.1 | 4.4 | 1.9 | 3.7 | 1.8 | 5.4 | 1.7 |
| Planning error | 23.0 | 6.1 | 14.6 | 3.5 | 34.3 | 7.7 | 30.9 | 8.2 |
| Spatial error | 14.1 | 5.9 | 48.2 | 7.5 | 20.2 | 7.0 | 15.3 | 6.5 |
| Time to completion | 32.9 | 4.8 | 32.2 | 3.9 | 29.2 | 3.5 | 25.5 | 2.1 |

**Supplementary Table S2.** Means and standard errors (SE) of $HbO_2$ concentration in children with and without an ASC during Lead, Follow, Turn-take, and Compete conditions of the Joint action task.

| **Region** | | **Lead** | | **Follow** | | **Turn-take** | | **Compete** | |
|---|---|---|---|---|---|---|---|---|---|
| | | **Mean** | **SE** | **Mean** | **SE** | **Mean** | **SE** | **Mean** | **SE** |
| | **TD group** | | | | | | | | |
| Left | MFG | 0.041 | 0.009 | 0.041 | 0.011 | 0.047 | 0.010 | 0.043 | 0.009 |
| | IFG | 0.073 | 0.012 | 0.071 | 0.014 | 0.078 | 0.014 | 0.077 | 0.013 |
| | PCG | 0.040 | 0.009 | 0.054 | 0.012 | 0.062 | 0.012 | 0.062 | 0.010 |
| | STS | 0.074 | 0.012 | 0.075 | 0.014 | 0.099 | 0.012 | 0.104 | 0.011 |
| | IPL | -0.023 | 0.009 | -0.012 | 0.011 | -0.043 | 0.008 | 0.004 | 0.011 |
| Right | MFG | 0.055 | 0.009 | 0.050 | 0.009 | 0.055 | 0.009 | 0.057 | 0.007 |
| | IFG | 0.081 | 0.011 | 0.067 | 0.013 | 0.063 | 0.013 | 0.063 | 0.012 |
| | PCG | 0.025 | 0.009 | 0.021 | 0.012 | 0.032 | 0.010 | 0.035 | 0.010 |
| | STS | 0.060 | 0.014 | 0.052 | 0.013 | 0.100 | 0.014 | 0.096 | 0.011 |
| | IPL | 0.029 | 0.012 | 0.016 | 0.012 | 0.022 | 0.012 | 0.023 | 0.009 |
| | ASC **group** | | | | | | | | |
| Left | MFG | 0.050 | 0.007 | 0.050 | 0.009 | 0.031 | 0.008 | 0.043 | 0.009 |
| | IFG | 0.057 | 0.011 | 0.080 | 0.012 | 0.052 | 0.010 | 0.076 | 0.012 |
| | PCG | 0.054 | 0.011 | 0.050 | 0.011 | 0.055 | 0.010 | 0.046 | 0.014 |
| | STS | 0.047 | 0.010 | 0.031 | 0.010 | 0.023 | 0.013 | 0.044 | 0.014 |
| | IPL | 0.059 | 0.010 | 0.012 | 0.009 | 0.007 | 0.007 | 0.005 | 0.010 |
| Right | MFG | 0.063 | 0.008 | 0.037 | 0.008 | 0.047 | 0.009 | 0.042 | 0.009 |
| | IFG | 0.060 | 0.010 | 0.070 | 0.012 | 0.042 | 0.010 | 0.062 | 0.010 |
| | PCG | 0.035 | 0.013 | 0.037 | 0.011 | 0.062 | 0.011 | 0.046 | 0.009 |
| | STS | 0.070 | 0.011 | 0.061 | 0.012 | 0.048 | 0.010 | 0.071 | 0.013 |
| | IPL | 0.036 | 0.011 | 0.008 | 0.012 | 0.023 | 0.009 | 0.060 | 0.014 |

**Supplementary Table S3.** Means and standard errors (SE) of HHb concentration in children with and without an ASC during Lead, Follow, Turn-take, and Compete conditions of the Joint action task.

| Region | | Lead | | Follow | | Turn-take | | Compete | |
|---|---|---|---|---|---|---|---|---|---|
| | | **Mean** | **SE** | **Mean** | **SE** | **Mean** | **SE** | **Mean** | **SE** |
| | **TD group** | | | | | | | | |
| Left | MFG | -0.026 | 0.003 | -0.027 | 0.004 | -0.019 | 0.003 | -0.020 | 0.003 |
| | IFG | -0.027 | 0.005 | -0.015 | 0.003 | -0.010 | 0.004 | -0.013 | 0.004 |
| | PCG | -0.039 | 0.006 | -0.038 | 0.004 | -0.028 | 0.004 | -0.038 | 0.005 |
| | STS | 0.043 | 0.006 | -0.050 | 0.004 | -0.056 | 0.006 | 0.044 | 0.005 |
| | IPL | -0.049 | 0.005 | -0.033 | 0.005 | -0.048 | 0.006 | -0.048 | 0.006 |
| Right | MFG | -0.036 | 0.004 | -0.033 | 0.003 | -0.035 | 0.003 | -0.025 | 0.003 |
| | IFG | -0.026 | 0.003 | -0.029 | 0.005 | -0.023 | 0.004 | -0.027 | 0.004 |
| | PCG | -0.055 | 0.005 | -0.042 | 0.005 | -0.049 | 0.006 | -0.058 | 0.007 |
| | STS | -0.049 | 0.006 | -0.060 | 0.007 | -0.040 | 0.006 | -0.054 | 0.005 |
| | IPL | -0.037 | 0.006 | -0.046 | 0.006 | -0.034 | 0.005 | -0.044 | 0.005 |
| | **ASC group** | | | | | | | | |
| Left | MFG | -0.048 | 0.004 | -0.052 | 0.007 | -0.035 | 0.005 | -0.049 | 0.006 |
| | IFG | -0.023 | 0.004 | -0.034 | 0.005 | -0.029 | 0.004 | -0.051 | 0.006 |
| | PCG | -0.052 | 0.005 | -0.052 | 0.006 | -0.045 | 0.006 | -0.061 | 0.007 |
| | STS | -0.069 | 0.009 | -0.061 | 0.006 | -0.050 | 0.005 | -0.074 | 0.009 |
| | IPL | -0.026 | 0.004 | -0.050 | 0.006 | -0.043 | 0.007 | -0.059 | 0.008 |
| Right | MFG | -0.059 | 0.007 | -0.072 | 0.008 | -0.037 | 0.005 | -0.057 | 0.007 |
| | IFG | -0.031 | 0.005 | -0.040 | 0.005 | -0.029 | 0.005 | -0.041 | 0.005 |
| | PCG | -0.081 | 0.008 | -0.090 | 0.010 | -0.060 | 0.006 | -0.086 | 0.009 |
| | STS | -0.067 | 0.006 | -0.063 | 0.008 | -0.062 | 0.007 | -0.078 | 0.007 |
| | IPL | -0.047 | 0.007 | -0.089 | 0.010 | -0.043 | 0.005 | -0.052 | 0.007 |

**Supplementary Table S4.** *p*-values and the effect directions of the significant post-hoc analyses for the 4-way interaction of Group × Condition × Hemisphere x Region for $HbO_2$ concentrations.

| **Comparison** | ***p*-values** | **Direction of effect** | **Effect sizes** |
|---|---|---|---|
| **Group related difference** | | | |
| Lead, left IPL | < 0.001 | ASC > TD[a] | -0.115 |
| Follow, left STS | 0.014 | TD > ASC[b] | -0.260 |
| Turn-take, left STS | < 0.001 | TD > ASC[a] | -0.154 |
| Turn-take, Left IPL | < 0.001 | ASC > TD[a] | -0.073 |
| Turn-take, Right PCG | 0.043 | ASC > TD[b] | -0.040 |
| Turn-take, Right STS | 0.003 | TD > ASC[a] | 0.058 |
| Compete, Left STS | 0.001 | TD > ASC[a] | 0.074 |
| Compete, Right IPL | 0.029 | ASC > TD[b] | -0.051 |
| **Condition related difference** | | | |
| TD, Left PCG | 0.013 | Compete > Lead[b] | -0.030 |
| TD, Left STS | 0.040 | Compete > Lead[b] | -0.031 |
| TD, Left IPL | 0.035 | Compete > Lead[b] | -0.038 |
| TD, Left IPL | 0.008 | Follow > Turn-take[b] | -0.095 |
| TD, Left IPL | 0.001 | Compete > Turn-take[a] | -0.068 |
| TD, Right IFG | 0.037 | Lead > Compete[b] | 0.022 |
| TD, Right STS | 0.013 | Turn-Take > Lead[b] | 0.536 |
| TD, Right STS | 0.004 | Compete > Lead[a] | 0.542 |
| TD, Right STS | 0.003 | Turn-take > Follow[b] | -0.055 |
| TD, Right STS | < 0.001 | Compete > Follow[a] | -0.050 |
| ASC, Left IFG | 0.027 | Follow > Turn-take[b] | 0.034 |
| ASC, Left IPL | < 0.001 | Lead > Follow[a] | 0.063 |
| ASC, Left IPL | < 0.001 | Lead > Turn-take[a] | 0.070 |
| ASC, Left IPL | < 0.001 | Lead > Compete[a] | 0.072 |
| ASC, Right MFG | 0.003 | Lead > Follow[a] | 0.037 |
| ASC, Right MFG | 0.042 | Lead > Compete[b] | 0.030 |
| ASC, Right IFG | 0.017 | Follow > Turn-take[b] | 0.034 |
| ASC, Right IPL | 0.006 | Lead > Follow[a] | 0.034 |
| ASC, Right IPL | < 0.001 | Compete > Follow[a] | -0.062 |
| ASC, Right IPL | 0.015 | Compete > Turn-take[b] | -0.050 |
| **Hemisphere related difference** | | | |
| TD, Lead, IPL | < 0.001 | Right > Left[a] | -0.073 |
| TD, Follow, PCG | 0.006 | Left > Right[b] | 0.040 |
| TD, Turn-take, PCG | 0.026 | Left > Right[b] | 0.036 |
| TD, Turn-take, IPL | < 0.001 | Right > Left[a] | -0.094 |
| TD, Compete, MFG | 0.026 | Right > Left[b] | -0.020 |
| TD, Compete, PCG | 0.011 | Left > Right[b] | 0.035 |
| ASC, Follow, STS | 0.007 | Right > Left[b] | -0.038 |
| ASC, Turn-take, MFG | 0.036 | Right > Left[b] | -0.024 |
| ASC, Turn-take, STS | 0.045 | Right > Left[b] | -0.028 |
| ASC, Compete, STS | 0.035 | Right > Left[b] | -0.030 |
| ASC, Compete, IPL | < 0.001 | Right > Left[a] | -0.072 |

[a]*p*-values which survived the FDR correction. [b]*p*-values < 0.05 which did not survive FDR correction.

**Supplementary Table S5.** *p*-values and the effect directions of the significant post hoc analyses for the 4-way interaction of Group × Condition × Hemisphere x Region for HHb concentrations.

| Comparison | *p*-values | Direction of effect | Effect sizes |
|---|---|---|---|
| **Group related difference** | | | |
| Lead | < 0.001 | TD > ASC[a] | 0.037 |
| Follow | < 0.001 | TD > ASC[a] | 0.077 |
| Turn-take | < 0.001 | TD > ASC[a] | 0.029 |
| Compete | < 0.001 | TD > ASC[a] | 0.076 |
| **Condition related difference** | | | |
| TD | 0.01 | Turn-take > Lead[b] | -0.015 |
| ASC | < 0.001 | Lead > Follow[a] | 0.029 |
| | 0.001 | Turn-take > Lead[b] | -0.021 |
| | <0.001 | Lead > Compete[a] | 0.029 |
| | <0.001 | Turn-take > Follow[a] | -0.046 |
| | <0.001 | Turn-take > Compete[a] | 0.053 |

[a]*p*-values which survived the FDR correction. [b]*p*-values < 0.05 which did not survive FDR correction.

**Supplementary Table S6.** Correlations between cortical activation and the behavioral errors in children with and without an ASC.

| ROIs | | TD group | | | ASC group | | |
|---|---|---|---|---|---|---|---|
| | | Motor | Planning | Spatial | Motor | Planning | Spatial |
| *Lead* | | | | | | | |
| Left | MFG | - | -0.113 | 0.079 | 0.149 | 0.098 | 0.146 |
| | IFG | - | -0.342** | -0.049 | 0.030 | -0.054 | -0.144 |
| | PCG | - | 0.435 | 0.086 | 0.012 | 0.020 | 0.295* |
| | STS | - | -0.259* | 0.079 | -0.119 | -0.094 | -0.142 |
| | IPL | - | -0.004 | -0.101 | 0.169 | 0.194 | 0.280 |
| Right | MFG | - | -0.215 | -0.117 | 0.191 | 0.141 | 0.062 |
| | IFG | - | -0.121 | 0.132 | 0.113 | -0.062 | -0.095 |
| | PCG | - | 0.056 | -0.094 | 0.278* | -0.013 | -0.082 |
| | STS | - | 0.075 | 0.004 | -0.060 | -0.234 | -0.059 |
| | IPL | - | -0.108 | 0.038 | 0.148 | -0.075 | -0.011 |
| *Follow* | | | | | | | |
| Left | MFG | -0.124 | -0.199 | -0.028 | 0.033 | 0.043 | -0.032 |
| | IFG | -0.132 | -0.323* | -0.186 | -0.183 | 0.016 | 0.029 |
| | PCG | -0.070 | -0.201 | -0.218 | -0.195 | 0.019 | -0.174 |
| | STS | -0.064 | -0.094 | -0.098 | -0.061 | 0.115 | -0.065 |
| | IPL | -0.184 | -0.223 | -0.081 | 0.016 | 0.005 | -0.033 |
| Right | MFG | 0.004 | -0.206 | -0.221 | -0.024 | 0.191 | 0.092 |
| | IFG | -0.139 | -0.098 | -0.191 | -0.028 | 0.097 | 0.115 |
| | PCG | -0.188 | 0.073 | -0.134 | 0.278* | 0.049 | 0.113 |
| | STS | -0.019 | 0.617 | -0.251 | -0.069 | -0.246 | -0.028 |
| | IPL | 0.102 | 0.066 | -0.167 | 0.002 | 0.007 | -0.126 |
| *Turn-take* | | | | | | | |
| Left | MFG | -0.207 | 0.068 | 0.101 | -0.081 | 0.075 | -0.040 |
| | IFG | -0.154 | -0.033 | 0.033 | -0.193 | 0.076 | -0.119 |
| | PCG | -0.162 | -0.066 | 0.284* | -0.067 | 0.150 | 0.096 |
| | STS | -0.019 | 0.049 | -0.093 | -0.100 | -0.220 | -0.021 |
| | IPL | -0.026 | 0.179 | -0.167 | 0.221 | 0.033 | -0.149 |
| Right | MFG | -0.184 | 0.120 | -0.069 | 0.002 | 0.256 | 0.108 |
| | IFG | 0.064 | 0.052 | -0.179 | 0.156 | -0.036 | 0.087 |
| | PCG | 0.004 | 0.157 | -0.070 | -0.007 | 0.268* | 0.109 |
| | STS | -0.041 | 0.238 | -0.094 | -0.030 | -0.052 | 0.024 |
| | IPL | -0.117 | -0.014 | -0.239 | -0.081 | -0.030 | -0.048 |
| *Compete* | | | | | | | |
| Left | MFG | -0.241 | -0.205 | -0.155 | -0.005 | -0.041 | -0.047 |
| | IFG | -0.113 | -0.276* | -0.209 | -0.028 | 0.090 | 0.007 |
| | PCG | -0.148 | -0.187 | -0.231 | -0.230 | 0.038 | -0.192 |
| | STS | -0.139 | 0.018 | 0.070 | -0.130 | -0.103 | -0.124 |
| | IPL | 0.086 | -0.094 | -0.118 | -0.140 | -0.038 | -0.172 |
| Right | MFG | -0.144 | -0.138 | -0.204 | -0.158 | -0.055 | -0.030 |
| | IFG | -0.086 | -0.062 | -0.064 | -0.033 | -0.056 | -0.002 |
| | PCG | -0.024 | -0.076 | -0.220 | -0.219 | -0.130 | 0.211 |
| | STS | -0.108 | 0.002 | -0.091 | -0.126 | -0.081 | -0.127 |
| | IPL | -0.088 | -0.053 | -0.021 | -0.102 | 0.096 | -0.176 |

* indicates $p < 0.05$; ** indicates $p < 0.01$. Bolded and shaded font indicates $p$ values survived the FDR corrections.

**Supplementary Table S7.** Spatial registration and assignment for right and left hemisphere channels.

| Side | CH | MNI coordinates | | | MFG | IFG | PCG | | STS | | IPL | | Other | | Assigned region |
|---|---|---|---|---|---|---|---|---|---|---|---|---|---|---|---|
| | | X | Y | Z | Middle frontal gyrus | Inferior frontal gyrus | Pre-central gyrus | Post-central gyrus | Superior temporal gyrus | Middle temporal gyrus | Supra-marginal gyrus | Angular gyrus | Superior frontal gyrus | Orbito-frontal gyrus | |
| Left | 7 | -34.3 | 41.3 | 42.3 | 95.9 | | | | | | | | 4.1 | | MFG |
| | 8 | -50.7 | 18.7 | 43.3 | 91.4 | | 8.6 | | | | | | | | MFG |
| | 9 | -60.0 | -7.7 | 44.3 | | | 39.7 | 60.3 | | | | | | | PCG |
| | 10 | -64.0 | -34.3 | 46.3 | | | | | | | 100.0 | | | | IPL |
| | 17 | -27.3 | 57.3 | 29.7 | 99.2 | | | | | | | | 0.8 | | MFG |
| | 18 | -48.0 | 37.3 | 30.3 | 99.6 | 0.4 | | | | | | | | | MFG |
| | 19 | -60.3 | 9.7 | 31.3 | 8.3 | 8.7 | 82.3 | 0.7 | | | | | | | PCG |
| | 20 | -67.0 | -19.7 | 33.3 | | | | 26.5 | | | 73.5 | | | | Excluded |
| | 21 | -65.0 | -46.3 | 36.7 | | | | | | | 66.7 | 33.3 | | | IPL |
| | 28 | -41.7 | 54.7 | 18.7 | 98.8 | 1.2 | | | | | | | | | MFG |
| | 29 | -57.0 | 27.7 | 18.7 | 6.3 | 93.7 | | | | | | | | | IFG |
| | 30 | -66.0 | -3.0 | 21.0 | | | 34.6 | 64.7 | | | 0.6 | | | | PCG |
| | 31 | -69.0 | -33.3 | 22.7 | | | | 0.3 | 49.8 | | 49.8 | | | | Excluded |
| | 38 | -29.3 | 67.0 | 4.7 | 99.3 | 0.7 | | | | | | | | | MFG |
| | 39 | -51.0 | 45.3 | 43.3 | 2.4 | 97.6 | | | | | | | | | IFG |
| | 40 | -59.7 | 15.3 | 7.3 | | 67.2 | 32.5 | | 0.3 | | | | | | IFG |
| | 41 | -69.0 | -17.3 | 6.7 | 15.3 | | | 14.6 | 70.1 | | | | | | STS |
| | 42 | -69.0 | -45.7 | 9.3 | | | | | 47.8 | 51.9 | 0.3 | | | | STS |
| | 49 | -40.7 | 59.3 | -8.3 | 15.5 | 49.4 | | | | | | | | 35.1 | Excluded |
| | 50 | -54.0 | 34.3 | -6.7 | | 54.8 | | | 3.7 | | | | | 41.5 | IFG |
| | 51 | -65.0 | -1.3 | -9.3 | | | | | 53.2 | 46.8 | | | | | STS |
| | 52 | -71.0 | -29.7 | -7.3 | | | | | | 100.0 | | | | | STS |
| Center / Undefined | 5 | 12.3 | 57.3 | 41.7 | 42.1 | | | | | | 57.9 | | | | Excluded |
| | 6 | -12.7 | 55.7 | 42.7 | 28.6 | | | | | | | | 71.4 | | Excluded |
| | 16 | 33.3 | 63.3 | 28.7 | | | | | | | | | 100 | | Excluded |
| | 26 | 14.7 | 71.0 | 19.3 | 75.3 | | | | | | | | 24.7 | | Excluded |
| | 27 | -16.7 | 70.0 | 18.7 | 59.3 | | | | | | | | 40.7 | | Excluded |
| | 37 | -2.7 | 70.7 | 5.7 | 1.3 | | | | | | | | 98.7 | | Excluded |
| | 47 | 14.7 | 72.7 | -7.3 | 65.7 | | | | | | | | 23.1 | 11.2 | Excluded |
| | 48 | -15.3 | 71.3 | -7.7 | 33.6 | | | | | | | | 56 | 10.4 | Excluded |
| Right | 1 | 67.0 | -29.0 | 45.0 | | | | | | | 99.7 | 0.3 | | | IPL |
| | 2 | 62.7 | -1.7 | 41.7 | | | 42.3 | 51.5 | | | 6.2 | | | | PCG |
| | 3 | 51.0 | 25.7 | 41.7 | 95.4 | 4.6 | | | | | | | | | MFG |
| | 4 | 33.7 | 46.7 | 40.7 | 100.0 | | | | | | | | | | MFG |
| | 11 | 68.0 | -40.3 | 35.3 | | | | | | | 62.4 | 37.6 | | | IPL |
| | 12 | 69.0 | -12.3 | 31.7 | | | | 54.8 | | | 45.2 | | | | Excluded |

| | | | | | | | | | | | | | | | |
|---|---|---|---|---|---|---|---|---|---|---|---|---|---|---|---|
| | 13 | 60.0 | 16.7 | 28.7 | 3.2 | 30.5 | 66.3 | | | | | | | | PCG |
| | 14 | 44.7 | 44.7 | 28.7 | 79.7 | 20.3 | | | | | | | | | MFG |
| | 15 | 25.3 | 61.7 | 29.3 | 100.0 | | | | | | | | | | MFG |
| | 22 | 71.0 | -26.3 | 21.3 | | | | 0.9 | 48 | | 45.2 | 5.8 | | | Excluded |
| | 23 | 67.0 | 46.7 | 19.7 | | 1.3 | 54.5 | 44.2 | | | | | | | PCG |
| | 24 | 56.3 | 36.7 | 17.7 | 0.4 | 99.6 | | | | | | | | | IFG |
| | 25 | 39.3 | 59.7 | 17.7 | 91.1 | 8.9 | | | | | | | | | MFG |
| | 32 | -69.0 | -33.3 | 22.7 | | | | | 33.5 | 66.5 | | | | | STS |
| | 33 | 71.0 | -10.3 | 46.7 | | | | 12.9 | 83.9 | 3.2 | | | | | STS |
| | 34 | 60.7 | 25.7 | 6.7 | | 83 | 17 | | | | | | | | IFG |
| | 35 | 49.3 | 52.7 | 4.3 | 6 | 94 | | | | | | | | | IFG |
| | 36 | 28.0 | 69.7 | 5.0 | 99.3 | 0.7 | | | | | | | | | MFG |
| | 43 | 73.0 | -22.7 | -8.7 | | | | | 2.5 | 97.5 | | | | | STS |
| | 44 | 64.3 | 5.7 | -10.3 | | | | | 54 | 46 | | | | | STS |
| | 45 | 54.7 | 42.7 | -7.3 | | 66.1 | | | | | | | | 33.9 | IFG |
| | 46 | 39.3 | 63.7 | -7.7 | 26.9 | 45.8 | | | | | | | | 27.3 | Excluded |

Undefined channels were located along the midline/center of the probe set and invariably overlapped between similar ROIs across the left and right hemispheres; hence were excluded. Unclear channels (grayed rows) were those channels and their homologues that did not clearly fall within a given ROI (<65% coverage within a given ROI).

**Supplementary Figure S1.** Picture card examples for the Lincoln Log Building Game. The building game consists of four brown logs and 4 color-coded supportive logs. The participating children were asked to build the logs according to the picture cards shown while playing leader/follower roles (Lead vs. Follow), while moving in synchrony or while taking turns (Lead/Follow vs. Turn-take), and moving cooperatively or competitively (Lead, Follow, and Turn-take vs. Compete) with adult partners.

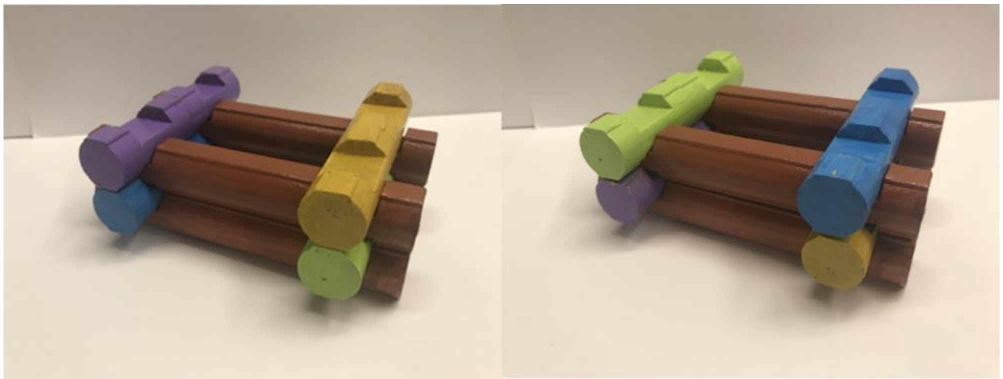

**Supplementary Figure S2.** The post hoc analyses for the condition x group 2-way interaction for HHb concentration. The group-related differences are presented in Supplementary Figure S2A, while the conditional differences in TD and ASC groups are presented in Supplementary Figure S2B and S2C, respectively. Children with ASC showed significantly more negative HHb concentration across all conditions compared to the TD group (all *p*s < 0.001, survived for FDR corrections, Supplementary Figure S2A). TD children showed similar level of HHb concentration (Supplementary Figure S2B) while children with ASC showed significantly differences in HHb concentration in different conditions (Lead > Follow; Lead > Compete; Turn-take > Follow; Turn-take > Compete; *p*s < 0.001, survived for FDR corrections, Supplementary Figure S2C).

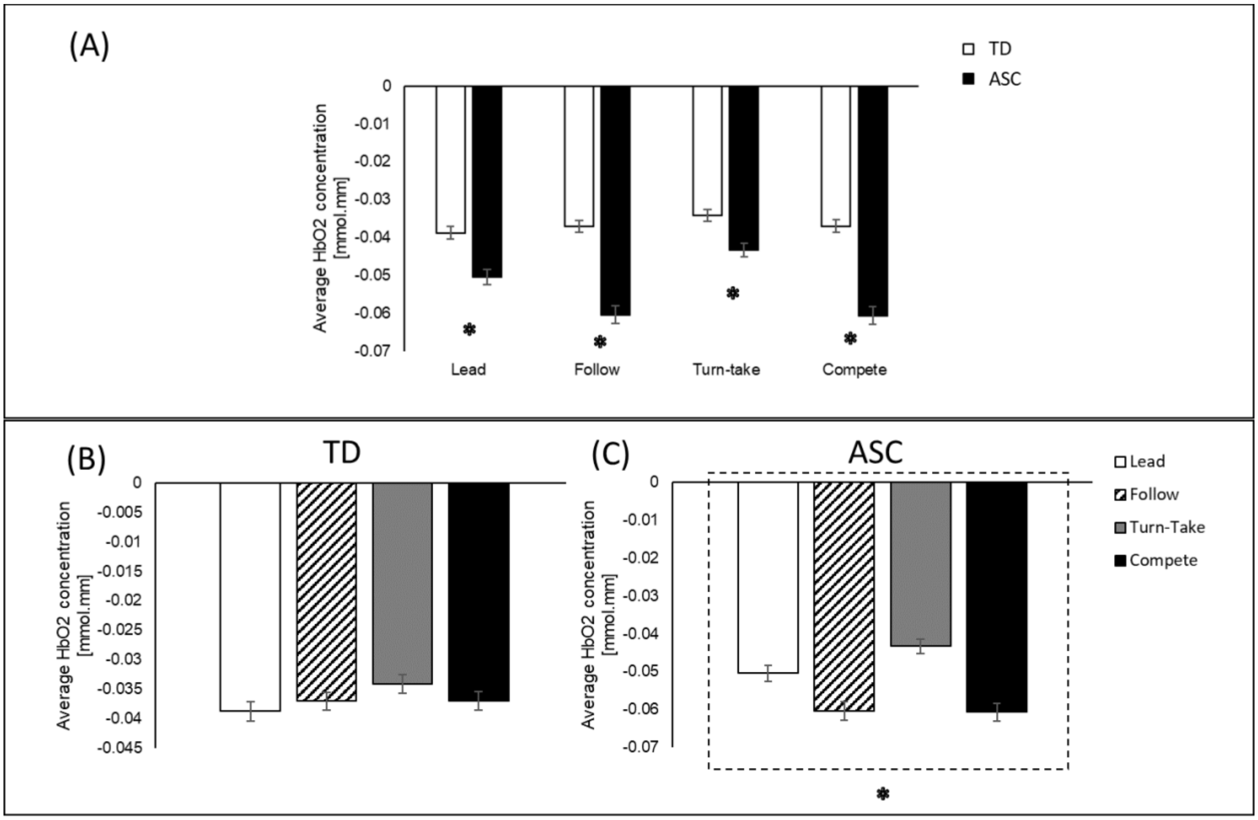

**Supplementary Figure S3.** Probe placement (A and B) and spatial registration output (C and D). Written permission has been taken for publication of participant pictures. MFG = middle frontal gyrus, IFG = inferior frontal gyrus; PCG = pre/post central gyrus; STS = superior temporal sulcus; IPL = inferior parietal lobe.

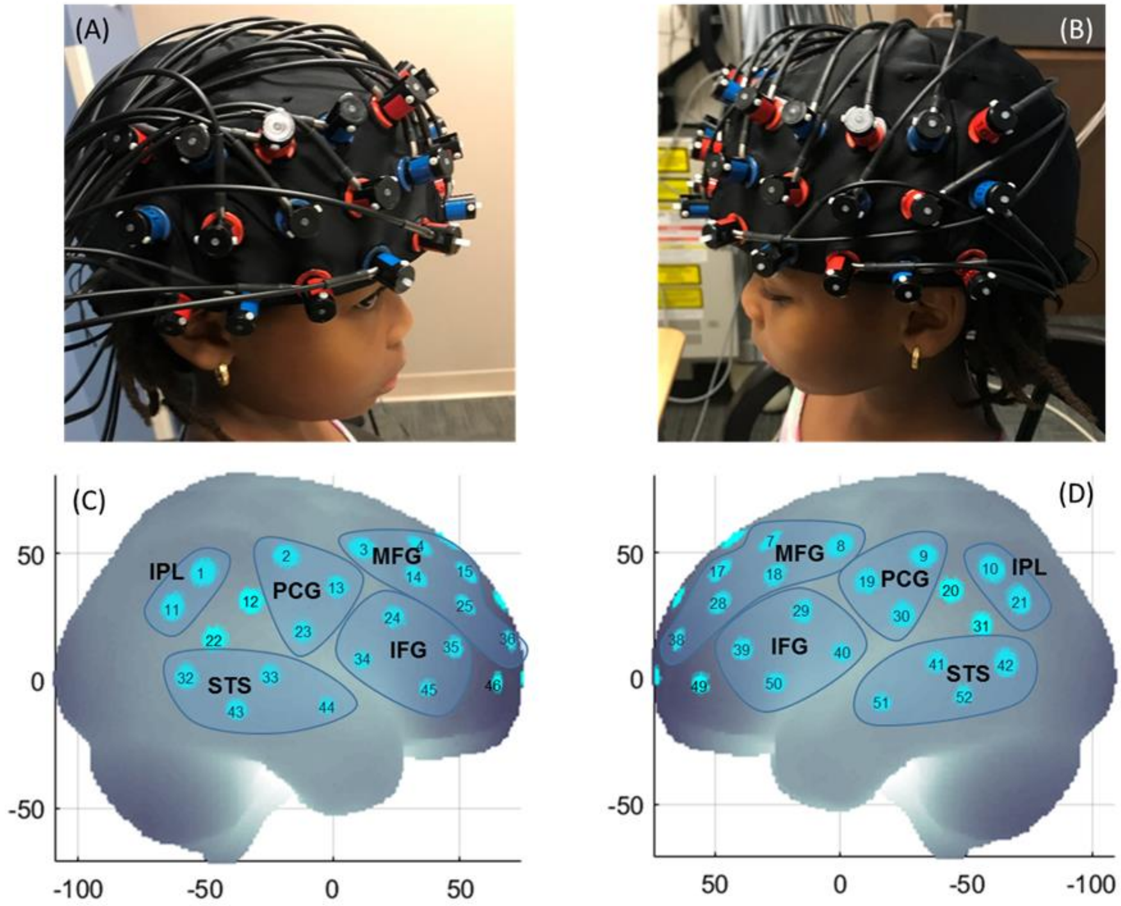

Supplement: Supplementary file 1 — Supplementary Information. [file 41598_2022_8689_MOESM1_ESM.pdf]
